# Advancing Patient Care: How Artificial Intelligence Is Transforming Healthcare

**DOI:** 10.3390/jpm13081214

**Published:** 2023-07-31

**Authors:** Diana Gina Poalelungi, Carmina Liana Musat, Ana Fulga, Marius Neagu, Anca Iulia Neagu, Alin Ionut Piraianu, Iuliu Fulga

**Affiliations:** 1 Saint Apostle Andrew Emergency County Clinical Hospital, 177 Brailei st., 800578 Galati, Romania; dianapoalelungi10@gmail.com (D.G.P.); mariusneagu87@gmail.com (M.N.); alin.piraianu@gmail.com (A.I.P.); fulgaiuliu@yahoo.com (I.F.); 2 Faculty of Medicine and Pharmacy, Dunarea de Jos University of Galati, 35 AI Cuza st., 800010 Galati, Romania; ancazanoschi@gmail.com; 3 ‘Saint John’ Clinical Emergency Hospital for Children, 800487 Galati, Romania

**Keywords:** artificial intelligence, machine learning, deep learning, clinical applications, digital pathology

## Abstract

Artificial Intelligence (AI) has emerged as a transformative technology with immense potential in the field of medicine. By leveraging machine learning and deep learning, AI can assist in diagnosis, treatment selection, and patient monitoring, enabling more accurate and efficient healthcare delivery. The widespread implementation of AI in healthcare has the role to revolutionize patients’ outcomes and transform the way healthcare is practiced, leading to improved accessibility, affordability, and quality of care. This article explores the diverse applications and reviews the current state of AI adoption in healthcare. It concludes by emphasizing the need for collaboration between physicians and technology experts to harness the full potential of AI.

## 1. Introduction

Artificial intelligence is increasingly being used as a virtual tool in many countries around the world. With its ability to mimic human cognitive functions, AI has revolutionized industries, improved efficiency, and unlocked new possibilities. During the past few years, governments have adopted a variety of smart applications that can use AI and its subsets provide predictions and recommendations in various fields, such as healthcare, finance, agriculture, education, social media, and data security.

Since the outbreak of COVID-19 in 2019, AI technologies have experienced accelerated adoption and utilization across various domains within the healthcare sector. In response to the pandemic, AI has emerged as a valuable tool and is being used for disease detection and diagnosis, medical imaging and analysis, treatment planning and personalized medicine, drug discovery and development, predictive analytics, and risk assessment. In 2018, Loh E. [1] stated that AI has the potential to significantly transform physicians’ roles and revolutionize the practice of medicine, and it is important for all doctors, in particular those in positions of leadership within the health system, to anticipate the potential changes, forecast their impact and make strategic plans for the medium to long term. In contrast, in 2021, Mistry C. et al. [2] assessed that the necessity for deploying advanced digital devices has become a requirement to offer augmented customer satisfaction, permitting tracking, checking the health status, and achieving better drug adherence.

**Figure 1:**
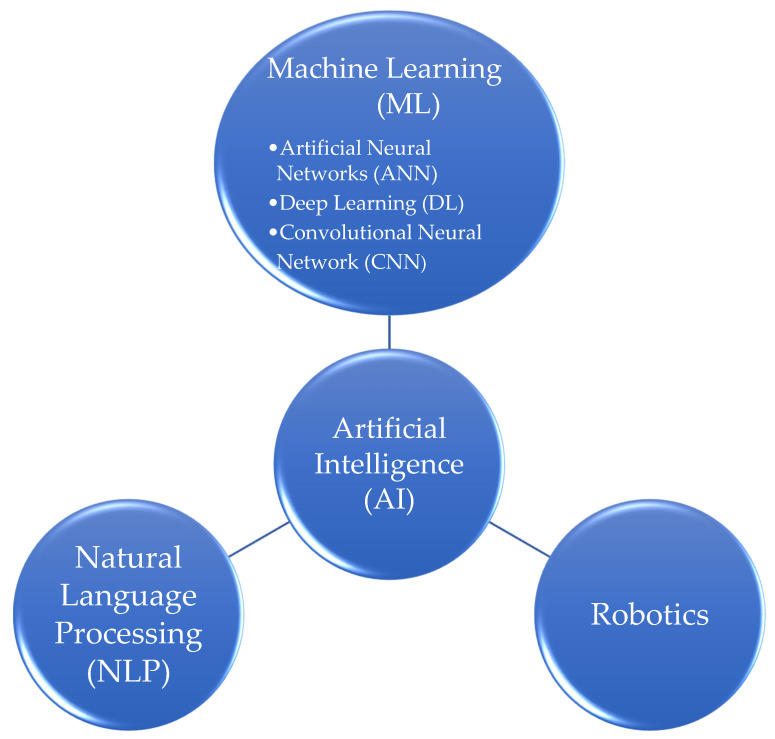
Illustration of the AI subtypes.

The field of AI is continuously evolving and researchers are exploring various avenues to create intelligent systems with different capabilities. The authors employed a visual representation, in the form of Figure 1, to illustrate the diverse subtypes of AI. Table 1 provides an overview of the definitions of terms related to AI and their integration within the healthcare sector.

## 2. Role of Artificial Intelligence in Healthcare

### 2.1. Disease Detection and Diagnosis and Medical Imaging

The application of AI within the diagnostic process supporting medical specialists could be of great value for the healthcare sector and the patients’ overall well-being [23]. The fundamental goal of the diagnosis of a disease lies in determining whether a patient is affected by a disease or not [24]. The first step in the diagnostic process involves obtaining a complete medical history and conducting a physical examination. For instance, a technique can use sound analysis to recognize COVID-19 from different respiratory sounds, e.g., cough, breathing, and voice [25]. Additionally, for a precise diagnosis, AI algorithms can be used for the analysis of medical scans and pathology images. Imaging applications include the determination of ejection fraction from echocardiograms [26], the detection and volumetric quantification of lung nodules from radiographs [27], and the detection and quantification of breast densities via mammography [28]. Imaging applications in pathology include an FDA-cleared system for whole-slide imaging (WSI) and their integration into a laboratory offers many benefits over light microscopy [29].

### 2.2. Treatment Planning and Personalized Medicine

AI tools have the ability to analyze large amounts of data and detect patterns. Therefore, they can make predictions for efficient and personalized treatment strategies. Personalized medicine, as an extension of medical sciences, uses practice and medical decisions to deliver customized healthcare services to patients [30]. For example, CURATE.AI is an AI-derived platform that maps the relationship between an intervention intensity (input-drug) and a phenotypic result (output) for an individual, based exclusively on that individual’s data, creating a profile, which serves as a map to predict the outcome for a specified input and to recommend the intervention intensity that will provide the best result [31].

### 2.3. Drug Discovery and Development

The use of AI has been increasing in the pharmaceutical industry, and as a result, it has reduced the human workload as well as achieved targets in a short period of time [32]. AI can recognize hit and lead compounds, and provide a quicker validation of the drug target and optimization of the drug structure design [33,34]. In January 2023, Insilico Medicine announced an encouraging topline readout of its phase 1 safety and pharmacokinetics trial of the molecule INS018_055, designed by AI for idiopathic pulmonary fibrosis, a progressive disease that causes scarring of the lungs [35].

### 2.4. Predictive Analytics and Risk Assessment

Disease risk assessment is the process of evaluating a person’s probability of developing certain diseases, based on risk factors such as genetic predispositions, environmental exposures, and lifestyle choices. AI techniques have been adopted to address the various steps involved in clinical genomic analysis—including variant calling, genome annotation, variant classification, and phenotype-to-genotype correspondence—and perhaps eventually they can also be applied to genotype-to-phenotype predictions [36]. Moreover, Ramazzotti et al. accomplished a successful prognosis prediction for 27 out of 36 cancers by employing AI to analyze various types of biological data such as RNA expression, point mutations, DNA methylation, and omics data of copy number variation. The data used for analysis was sourced from The Cancer Genome Atlas (TCGA) [37].

## 3. Literature Review

### 3.1. Methodology

We conducted a comprehensive review of current literature including original articles that studied various clinical applications of AI in healthcare. We performed extensive searches on Google Scholar, PubMed, and ScienceDirect databases to identify relevant manuscripts. As keywords, we used “artificial intelligence”, “deep learning”, and “machine learning”, combined with “clinical applications”, and “healthcare”. We restricted our search to papers published in English between 2013 and 2023 and found more than 200 relevant manuscripts. The inclusion criteria focused on studies that examined the application of artificial intelligence in different medical specialties. We excluded reviews and editorial comments.

### 3.2. Results

After a thorough review and assessment of the 223 articles, we identified and included a subset of 52 papers that were directly relevant to our research, including four on cardiology, three on dermatology, two on gastroenterology, three on neurology and neuroscience, three on ophthalmology, three on psychiatry, three on forensics and toxicology, four on radiology, 17 on pathology, two on urology, and four on obstetrics and gynecology, listed in Table 2. These selected studies provided valuable insights into the use and impact of AI in various medical specialties, forming the basis of our review.

#### 3.2.1. AI in Cardiology

As Attia Z.I. et al. (2019) and Alsharqi M. et al. (2018) declared, using machine learning and deep learning, AI has been deployed to interpret echocardiograms, to automatically identify heart rhythms from an electrocardiogram (ECG), to uniquely identify an individual using the ECG as a biometric signal, and to detect the presence of heart disease such as left ventricular dysfunction from the surface ECG [38,39,40]. In a study conducted in China by Weng S.F. et al. between 2005 and 2015, using routine clinical data of over 350,000 patients, machine learning significantly improved the accuracy of cardiovascular risk prediction, correctly predicting 355 (an additional 7.6%) more patients who developed cardiovascular disease compared with the established algorithm [41].

#### 3.2.2. AI in Dermatology

According to Young AT. et al. (2020), automated AI diagnosis of skin lesions is ready to be tested in clinical environments and has the potential to provide diagnostic support and expanded access to care [42]. A meta-analysis of 70 studies found the accuracy of computer-aided diagnosis of melanoma to be comparable to that of human experts [43]. In 2017, Esteva et al. supported the view that a convolutional neural network (CNN), the leading DL algorithm for image analysis, trained on 129,450 images, achieved performance comparable to dermatologists on two binary classification tasks, carcinomas versus seborrheic keratoses and melanomas versus nevi, for both dermoscopic and non-dermoscopic images [44]. 

#### 3.2.3. AI in Gastroenterology

Kröner PT. et al. (2021) stated that the clinical applications of AI systems in gastroenterology and hepatology include the identification of premalignant or malignant lesions (e.g., identification of dysplasia or esophageal adenocarcinoma in Barrett’s esophagus, pancreatic malignancies), detection of lesions (e.g., polyp identification and classification, small-bowel bleeding lesion on capsule endoscopy, pancreatic cystic lesions), development of objective scoring systems for risk stratification, predicting disease prognosis or treatment response (e.g., determining survival in patients post-resection of hepatocellular carcinoma), determining which patients with inflammatory bowel disease (IBD) will benefit from biologic therapy, or evaluation of metrics such as bowel preparation score or quality of endoscopic examination [45]. A study conducted by Martin D.R. et al. (2020) using histopathologic images of gastric biopsies as an input had a diagnostic accuracy of 98.9–99.1% for detecting current Helicobacter pylori infection vs. 79.0–79.4% mean accuracy of endoscopists for detecting currently infected H. pylori in two studies [46].

#### 3.2.4. AI in Neurology and Neuroscience

According to Pedersen M. (2020), AI has the potential to create a paradigm shift in the diagnosis, treatment, prediction, and economics of neurological disease [47]. Hazlett HC. et al. (2017) stated that a deep learning algorithm used magnetic resonance imaging (MRI) of the brain of individuals 6 to 12 months old to predict the diagnosis of autism in individual high-risk children at 24 months, with a positive predictive value of 81% [48]. Moreover, Ienca M. and Ignatiadis K. (2020) emphasized that the use of pattern recognition and feature extraction algorithms, for example, can significantly contribute to diagnosing brain diseases, such as brain tumors or Alzheimer’s disease, earlier, more accurately, and at more treatable stages compared to conventional predictive models [49].

#### 3.2.5. AI in Ophthalmology

Rathi S. et al. (2017) declared that teleophthalmology has been well established to aid in the detection of retinopathy of prematurity (ROP), diabetic retinopathy screening, and is being explored for glaucoma screening and other fields of ophthalmology [50]. Furthermore, Gulshan V. et al. (2016) demonstrated the clinical utility of a deep machine-learning algorithm that evaluated retinal fundus photographs from adults that detected referable diabetic retinopathy with high sensitivity and specificity [51]. Long E. et al. (2017) showed that an AI agent, using deep learning and neural networks, accurately diagnosed and provided treatment decisions for congenital cataracts in a multihospital clinical trial, performing just as well as individual ophthalmologists [52].

#### 3.2.6. AI in Psychiatry

The emerging literature has shown that AI is proving to be useful in psychological medicine and psychiatry. According to Pham KT. et al. (2022), within the last two decades, AI began to incorporate neuroimaging studies of psychiatric patients with deep learning models to classify patients with psychiatric disorders [53]. Vieira S. et al. (2017) were able to classify schizophrenia patients and controls with an accuracy of 85.5% by extracting functional connectivity patterns from resting-state functional MRIs of schizophrenia patients and healthy controls [54]. Researchers at the Vanderbilt University Medical Centre created machine-learning algorithms that achieved 80–90% accuracy when predicting whether someone will attempt suicide within the next 2 years, and 92% accuracy in predicting whether someone will attempt suicide within the next week [1].

#### 3.2.7. AI in Forensics and Toxicology

Forensic medicine and toxicology are important branches of the investigation of crimes. In 2022, Wankhade TD. et al. stated that various procedures of forensic medicine such as analysis of toxins, collection of the various samples of medicolegal importance from body cavities, detection of pathological changes in various organs of the body, detection of various stains on the body, detection of a weapon used in crime, time since death calculations, etc. are the areas where AI will play a key role in framing the various opinions of medicolegal importance [55]. For example, according to Thurzo A. et al. (2021), three-dimensional convolutional neural networks (3D CNN) of artificial intelligence can be used in biological age determination, sex determination, automatized 3D cephalometric landmark annotation, soft-tissue face prediction from the skull and in reverse, and facial growth vectors prediction [56].

In toxicology, deep learning might automatically identify high-level drug use patterns by combining data from social media, poison control logs, published reports, and national surveys [57].

#### 3.2.8. AI in Radiology

According to Hosny A. et al. (2018), AI methods automatically recognize complex patterns in imaging data and provide quantitative, rather than qualitative, assessments of radiographic characteristics [58]. Chen, H et al. (2016) maintained that studies have also shown that deep learning technologies are on par with radiologists’ performance for both detection [59] and segmentation [60] tasks in ultrasonography and MRI, respectively. Additionally, Wang, H. et al. (2017) declared that for the classification tasks of lymph node metastasis in PET–CT (positron emission tomography-computed tomography), deep learning had higher sensitivities but lower specificities than radiologists [61].

#### 3.2.9. AI in Surgery

According to Zhou, XY. et al. (2020), advances in surgery have revolutionized the management of both acute and chronic diseases, prolonging life and extending the boundary of patient survival [62]. Moreover, current robots can already automatically perform some simple surgical tasks, such as suturing and knot tying [63,64]. For example, in 2016, a smart surgical robot stitched up a pig’s small intestines completely on its own and was able to outperform human surgeons who were given the same task [65].

#### 3.2.10. AI in Pathology

In the modern healthcare system, AI and Digital Pathology (DP) have the potential to challenge traditional practice and provide precision for pathology diagnostics. Cui M., and Zhang D.Y. (2021) defined DP as the process of digitizing histopathology, immunohistochemistry, or cytology slides using whole-slide scanners as well as the interpretation, management, and analysis of these images using computational approaches [66]. According to Niazi M. K. K. et al. (2019), whole-slide imaging (WSI) allows entire slides to be imaged and permanently stored at high resolution, a process that provides a vast amount of information, which can be shared for clinical use or telepathology [67]. Two scanners, the Philips IntelliSite Pathology Solution (PIPS) and Leica Aperio AT2 DX, are approved by the Food and Drug Administration (FDA) to review and interpret digital surgical pathology slides prepared from biopsied tissue [68,69].

The use of digital image analysis in pathology can identify and quantify specific cell types quickly and accurately and can quantitatively evaluate histological features, morphological patterns, and biologically relevant regions of interest [72,73,74]. As Balázs et al. (2020) declared, recent groundbreaking results have demonstrated that applications of machine learning methods in pathology significantly improve Ki67 scoring in breast cancer, Gleason grading in prostate cancer, and tumor-infiltrating lymphocyte (TIL) scoring in melanoma [74]. Shaban et al. (2019) trained a novel CNN system to quantify TILs from WSIs of oral squamous cell carcinomas and achieved an accuracy of 96% [75]. Furthermore, Hekler A. et al. conducted a study in 2019 which concluded that a CNN was able to outperform 11 histopathologists in the classification of histopathological melanoma images and thus shows promise to assist human melanoma diagnoses [76]. Table 3 summarize the applications of AI systems in pathology.

In 2014, Dong et al. designed a computational pathology method to identify and quantify nuclear features from diagnostic tumor regions of interest (ROIs) of intraductal proliferative lesions of the breast, with high accuracy for distinguishing between benign breast ductal hyperplasia and malignant ductal carcinoma in situ [77]. Moreover, Coutre et al. (2018) used image analysis with DL to detect breast cancer histologic subtypes [80]. In addition, AI algorithms have been developed to provide quantitative measurements of immunohistochemically stained Ki-67 [81], ER [80], PR, and Her-2/neu images [82].

#### 3.2.11. AI in Urology

AI applications in urology include: utilizing radiomic imaging or ultrasonic echo data to improve or automate cancer detection or outcome prediction, utilizing digitized tissue specimen images to automate detection of cancer on pathology slides, and combining patient clinical data, biomarkers, or gene expression to assist disease diagnosis or outcome prediction [89]. For example, Kott et al. tested an AI-based system for detecting prostate cancer which yielded 91.5% accuracy in classifying slides as either benign or malignant, and 85.4% accuracy in finer classifications of benign vs. Gleason 3 vs. 4 vs. 5 [83]. In another study, Baessler et al. applied ML-based CT radiomics to determine whether the lymph nodes dissected in patients with metastatic or advanced non-seminomatous testicular germ cell tumor were benign or malignant, with 88% sensitivity, and 72% specificity [84]. 

#### 3.2.12. AI in Obstetrics and Gynecology

##### AI in Obstetrics

The fields of prenatal diagnosis, labor, and high-risk pregnancy are areas of significant importance in medicine, and they can be associated with medicolegal issues. Studies show that AI tools can be used to reduce these issues and to improve patients’ outcomes (both mothers’ and newborns’). In a study conducted by Idowu et al. [85], electrohysterography signals were employed, and three distinct machine learning algorithms were utilized to assist in the accurate detection of true labor, and the reliable diagnosis of premature labor. In another study, Manna et al. [86] proposed a method that combines AI and ANNs to extract texture descriptors from oocyte or embryo images. This approach enables AI to effectively identify the most viable oocytes and embryos, increasing the likelihood of successful pregnancies.

##### AI in Gynecology

Numerous research investigations focusing on cervical cancer and cervical intraepithelial neoplasia (CIN) have documented the application of AI. The primary areas where AI has been employed include the assessment of colposcopy, MR imaging (MRI), CT scans, cytology, and data related to human papillomavirus (HPV) [90]. Additionally, Zhang et al. [87] demonstrated in their research that using deep learning on color ultrasound tests as imaging assessments resulted in an impressive accuracy of 0.99 in predicting the definitive diagnosis of ovarian tumors. Moreover, Hart G. et al. emphasized that the application of machine learning shows immense potential in aiding the early detection of endometrial cancer. This approach achieves high-accuracy predictions by primarily relying on personal health information even before the onset of the disease, eliminating the necessity for invasive or costly procedures such as endometrial biopsy [88].

## 4. Discussion and Challenges

The literature review underscores the remarkable potential of AI in different medical specialties, to revolutionize screening and diagnostic procedures, and therefore, improving patient care. AI can improve diagnostic accuracy while limiting errors and impact patient safety such as assisting with prescription delivery [91,92,93]. Nevertheless, there are some challenges that need to be considered as AI usage increases in healthcare, such as ethical, social and technical challenges. For example, AI processes may lack transparency, making accountability problematic, or may be biased, leading to unfair, discriminatory behavior or mistaken decisions [94]. Moreover, AI algorithms are unable to perform a holistic approach to clinical scenarios and are not fully able to take into consideration the psychological and social aspects of human nature, which are often considered by a skilled healthcare professional [95]. Addressing those challenges requires collaboration between healthcare professionals, researchers, policymakers and technology developers to ensure that AI tools are implemented responsibly, ethically and safely in the healthcare sector.

## 5. Conclusions

Artificial intelligence systems powered by machine learning and deep learning are rapidly implemented in medicine. Moreover, combining AI with actual knowledge in various medical specialties could result in dramatic changes, such as advanced diagnostics, correct risk and prognosis evaluation, and even treatment suggestions. Thus far, AI is proving to be effective and the research will continue to improve, as more applications are discovered and explored. The integration of digital pathology based on AI systems in our current practice will help enhance patient care. In conclusion, AI’s role in medicine will continue to expand. In collaboration with experts in technology and ethics, we can revolutionize health care, making it more precise and we can pave the way for a healthier future with the right implementations of AI.

## Figures and Tables

**Table 1:** Definitions of terms related to AI.

Term	Definition
Artificial Intelligence (AI)	The first definition was been given in 1950 by Alan Turing, the founding father of AI, as the science and engineering of making intelligent machines, especially intelligent computer programs [3]. According to Salto-Tellez M. et al. [4], AI represents a range of advanced machine technologies that can derive meaning and understanding from extensive data inputs, in ways that mimic human capabilities. In the present context of medical practice, a specific definition may be a system’s ability to correctly interpret external data, to learn from such data, and to use those learnings to achieve specific goals and tasks through flexible adaptation [5].
Machine Learning (ML)	ML, a subset of artificial intelligence, exhibits the experiential “learning” associated with human intelligence, while also having the capacity to learn and improve its analyses through the use of computational algorithms [6,7]. Alpaydin E. [8] defined machine learning as the field of programming computers to optimize a performance criterion using example data or past experience. ML-based tools are used in the healthcare system to provide various treatment alternatives and individualized treatments and improve the overall efficiency of hospitals and healthcare systems while lowering the cost of care [9].
Deep Learning (DL)	Deep Learning, a subset of Machine Learning, refers to a deep neural network, which is a specific configuration where neurons are organized in multiple successive layers that can independently learn representations of data and progressively extract complex features, performing tasks such as computer vision and natural language processing (NLP) [10]. In experimental settings across multiple medical specialties, DL performs equivalently to healthcare professionals for detecting diseases from medical imaging [11].
Natural Language Processing (NLP)	Natural Language Processing is a theoretically-motivated range of computational techniques for analyzing and representing naturally-occurring texts at one or more levels of linguistic analysis for the purpose of achieving human-like language processing for a range of tasks or applications [12]. NLP techniques have been used to structure information in healthcare systems by extracting relevant information from narrative texts so as to provide data for decision-making [13].
Robotics	The robot has been defined as “a reprogrammable multifunctional manipulator designed to move material, parts, tools, or specialized devices through variable programmed motions for the performance of a variety of tasks” by the Robot Institute of America [14]. The term “robotics” refers to the study and use of robots. Robotic assistance has been shown to improve the safety and performance of intracorporeal suturing, which is heavily required in urological and gynecological procedures [15].
Artificial Neural Network (ANN)	An Artificial Neural Network, a subset of Machine Learning, is a computational model inspired by the biological neural networks in the human brain. These systems are mainly used for pattern identification and processing and are able to progressively improve their performance based on analytic results from previous tasks [16,17,18]. Many areas have been integrating the use of ANNs to facilitate the diagnosis, prognosis, and treatment of many diseases [19,20,21].
Convolutional Neural Network (CNN)	A Convolutional Neural Network is a Deep Learning algorithm specifically designed for image and video processing, primarily used in medical image analysis and diagnostics. CNNs have demonstrated superior performance as compared with classical machine learning algorithms and in some cases achieved comparable or better performance than clinical experts [22].

**Table 2:** Scientific articles that analyze the use of artificial intelligence in medical specialties.

Medical Specialty	Year of Study	Author	Application
Cardiology	2019	Attia Z.I. [38]	Screening for cardiac contractile dysfunction
	2019	Attia Z.I. [39]	Detection of left ventricular systolic dysfunction
	2018	Alsharqi M. [40]	Echocardiography analysis
	2017	Weng S.F. [41]	Cardiovascular risk prediction
Dermatology	2020	Young A.T. [42]	Diagnosis of skin lesions
	2019	Dick V. [43]	Diagnosis of melanoma
	2017	Esteva A. [44]	Classification of skin cancer
Gastroenterology	2021	Kröner P.T. [45]	Detection of various lesions
	2020	Martin D.R. [46]	Detecting current Helicobacter pylori infection
Neurology and Neuroscience	2020	Pedersen M. [47]	Diagnosis of neurological diseases
	2017	Hazlett H.C. [48]	Diagnosis of autism
	2020	Ienca M. [49]	Diagnosis of Alzheimer’s disease
Ophthalmology	2017	Rathi S. [50]	Teleophthalmology for retinopathy and glaucoma
	2016	Gulshan V. [51]	Detection of diabetic retinopathy
	2017	Long E. [52]	Diagnosis of congenital cataracts
Psychiatry	2022	Pham K.T. [53]	Classification of psychiatric disorders
	2017	Vieira S. [54]	Classification of schizophrenia patients
	2018	Loh E. [1]	Prediction of suicide attempts
Forensics and Toxicology	2022	Wankhade T.D. [55]	Detection of various samples
	2021	Thurzo A. [56]	Identification of a cadaver
	2020	Chary M.A. [57]	Identification of drug use patterns
Radiology	2018	Hosny A. [58]	Recognition of complex radiographic patterns
	2016	Chen H. [59]	Detection in ultrasonography
	2017	Ghafoorian M. [60]	Segmentation in magnetic resonance imaging (MRI)
	2017	Wang H. [61]	Classification of mediastinal lymph node metastasis
Surgery	2020	Zhou X.Y. [62]	Advances in surgery
	2018	Hu Y. [63]	Robotic sewing and knot tying
	2019	Hu Y. [64]	Suturing robot for transanal endoscopic microsurgery
	2016	Shademan A. [65]	Robotic soft tissue surgery
Pathology	2021	Cui M. [66]	Digitizing histopathology
	2019	Niazi M.K.K. [67]	Whole-slide imaging
	2017	FDA [68]	IntelliSite Pathology Solution
	2019	FDA [69]	Summary Aperio AT2 DX system
	2017	Araújo T. [70]	Classification of breast cancer
	2017	Tumeh P.C. [71]	Identification of the immune cell populations
	2019	Bera K. [72]	Quantitative evaluation of histological and morphological patterns
	2018	Mezheyeuski A. [73]	Classification of lung cancer patients
	2020	Balázs A. [74]	Detection of metastasis and micrometastasis
	2019	Shaban M. [75]	Prediction of disease-free survival in oral squamous cell carcinoma
	2019	Hekler A. [76]	Classification of histopathological melanoma images
	2014	Dong F. [77]	Distinction between benign and malignant intraductal proliferations of the breast
	2015	Veta M. [78]	Mitosis detection in breast cancer
	2013	Cireşan D.C. [79]	Mitosis detection in breast cancer
	2018	Couture H.D. [80]	Prediction of breast cancer grade
	2018	Sahiner B. [81]	Application to Ki67 staining
	2019	Hossain M.S. [82]	Automatic quantification of *HER2* gene amplification
Urology	2021	Kott O. [83]	Diagnosis of prostate cancer and Gleason grading
	2020	Baessler B. [84]	Detection of metastatic testicular germ cell tumors
Obstetrics and Gynecology	2015	Idowu I. [85]	Detection of true labor and diagnosis of premature labor
	2013	Manna C. [86]	Identification of most viable oocytes and embryos
	2019	Zhang L. [87]	Diagnosis of ovarian tumor
	2020	Hart G. [88]	Early detection of endometrial cancer

**Table 3:** Examples of AI systems applications in pathology.

Examples of AI Systems Applications in Pathology
1. Differentiate between benign and malignant tumors
2. Grading of dysplasia and in situ lesions [70]
3. Metastasis and micrometastasis detection [74]
4. Relationships between different immune cell populations [70,71]
5. IHC/ISH scoring of multiple biomarkers and topography of the immune response [72]
6. Mitosis detection [78,79]

## Data Availability

Not applicable.
